# Potentials of Curcumin as an Antidepressant

**DOI:** 10.1100/tsw.2009.137

**Published:** 2009-11-01

**Authors:** S.K. Kulkarni, Ashish Dhir, Kiran Kumar Akula

**Affiliations:** Pharmacology Division, University Institute of Pharmaceutical Sciences, Panjab University, Chandigarh, India

**Keywords:** curcumin, depression, animal models, neurotrophic factors, neurotransmitters, inflammation

## Abstract

Major depression, a debilitating psychiatric disorder, is predicted to be the second most prevalent human illness by the year 2020. Various antidepressants, ranging from monoamine oxidase inhibitors to recently developed dual reuptake inhibitors, are prescribed for alleviating the symptoms of depression. Despite the availability of these blockbuster molecules, approximately 30% of depressed patients do not respond to the existing drug therapies and the remaining 70% fails to achieve complete remission. Moreover, antidepressants are associated with a plethora of side effects and drug-drug/drug-food interactions. In this context, novel approaches are being tried to find more efficacious and safer drugs for the treatment of major depression. Curcumin is one such molecule that has shown promising efficacy in various animal models of major depression. Although the mechanism of the antidepressant effect of curcumin is not fully understood, it is hypothesized to act through inhibiting the monoamine oxidase enzyme and modulating the release of serotonin and dopamine. Moreover, evidences have shown that curcumin enhances neurogenesis, notably in the frontal cortex and hippocampal regions of the brain. The use of curcumin in clinics for the treatment of major depression is limited due to its poor gastrointestinal absorption. The present review attempts to discuss the pharmacological profile along with molecular mechanisms of the antidepressant effect of curcumin in animal models of depression. A need for clinical trials in order to explore the antidepressant efficacy and safety profile of curcumin is emphasized.

## INTRODUCTION

Curcumin, the main curcuminoid found in the spice turmeric, is a plant alkaloid obtained from *Curcuma longa* (Fam.: Zingiberaceae). It has been used in the Indian and Chinese systems of medicines to treat wounds and sprains, and gastrointestinal, pulmonary, and liver disorders[1]. Many pharmacological studies have been conducted to describe multiple biological actions of curcumin. These studies have demonstrated that curcumin possesses antioxidant[2], anti-inflammatory[3,4,5], anticarcinogenic[6], antimicrobial[7,8,9], hepatoprotective[8], hypoglycemic[10,11,12], thrombosuppressive[13], and antiarthritic[14] activities. Curcumin has also demonstrated neuroprotective effects in animal models of

Alzheimer's disease[15], Parkinson's disease[16], schizophrenia[17], drug addiction[18], Prion's infection[19], stroke[20], aluminum neurotoxicity[21], epilepsy[22], and diabetic neuropathy[23] (Fig. 1). There has been an exponential increase in the trend of research involved in exploring the efficacy of curcumin in animal models of major depression during last few years (Fig. 2).

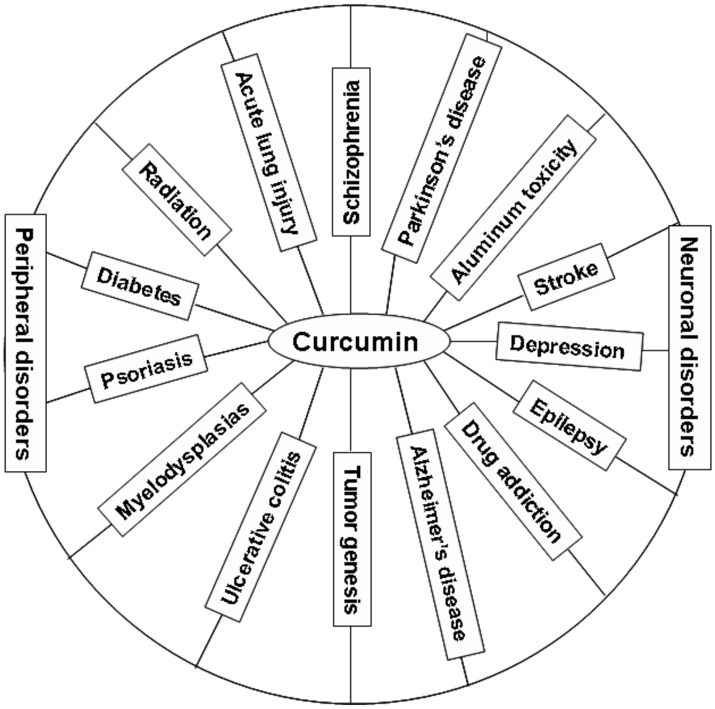

**FIGURE 1.** Therapeutic effects of curcumin in several human disorders.

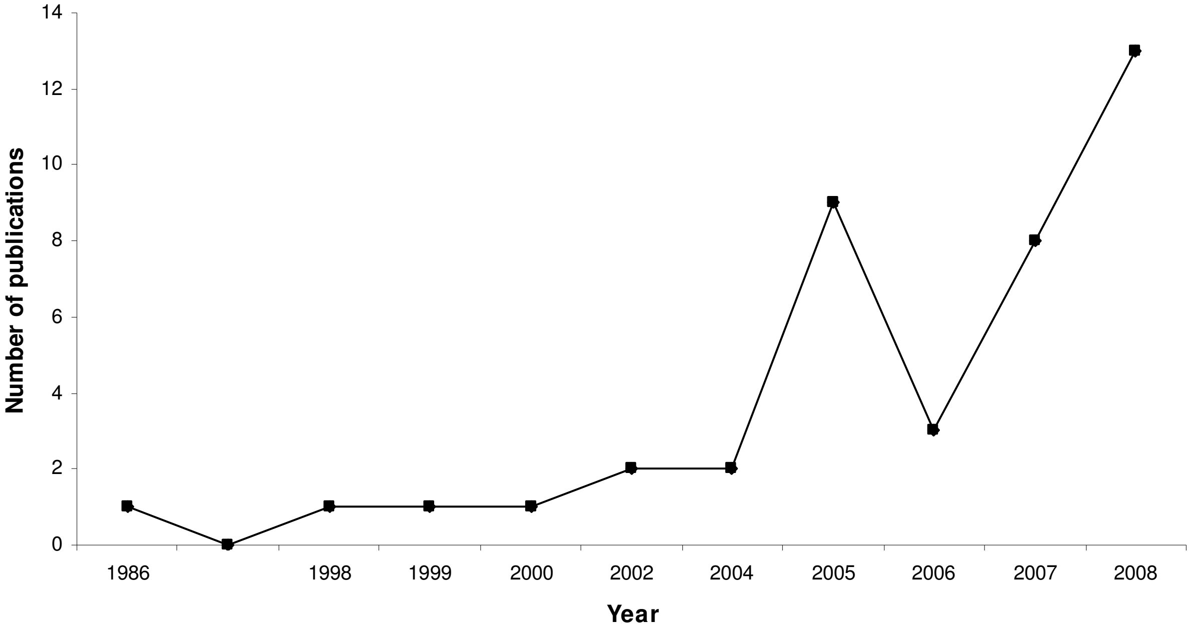

**FIGURE 2.** Scopus hits using curcumin and depression as keywords. Source: www.scopus.com

Major depression represents the most common and proliferating health problem worldwide[24,25], and has an estimated lifetime prevalence of about 15–20%[26,27,28]. The common symptoms of major depression include depressed or irritable mood, decreased interest in pleasurable activities, significant weight loss or gain, insomnia or hypersomnia, psychomotor agitation or retardation, fatigue or loss of energy, feeling of worthlessness or excessive guilt, decreased concentrating power, and increase in suicidal tendencies. Earlier, major depression was considered to be an old-age disease. However, current trends reveal an increased percentage of younger populations being affected from its consequences. Major depression is relatively common among patients with a diagnosis of dementia[29,30] and also may pose a risk factor for development of dementia[31]. Epidemiological studies have revealed that people consuming curcumin in daily life have sharper brain functions and higher cognitive abilities. Curcumin possesses some interesting properties that justify its use in major depression. These include:

- **Curcumin is an inhibitor of monoamine oxidase (MAO) enzyme:** The MAO enzyme is expressed on the outer membrane of mitochondria in most of the body's cells, where it is involved in catalyzing the oxidation of monoamines. It is known to exist in two isoforms, MAO-A and MAO-B. MAO-B is the predominant form of the enzyme in the human brain and oxidizes dopamine, whereas norepinephrine and serotonin are the preferred substrates for MAO-A[32]. Interestingly, curcumin possesses both MAO-A– and MAO-B–inhibiting properties[32].
- **Curcumin modulates the level of various neurotransmitters:** Curcumin modulates the levels of norepinephrine, dopamine, and serotonin in the brain[32,33]. Norepinephrine is a neurotransmitter involved in attentiveness, emotions, sleeping, dreaming, and learning. Dopamine is involved in pleasure, emotion, and regulating locomotion, while serotonin has a major role to play in neurovegetative functions of the body, such as appetite, sleep, memory and learning, temperature regulation, mood, behavior (including sexual and hallucinogenic behavior), cardiovascular functions, muscle contraction, and endocrine regulation.
- **Curcumin promotes hippocampal neurogenesis:** Curcumin has demonstrated an ability to increase the levels of brain-derived neurotrophic factor (BDNF). In one of the studies carried out by Xu and colleagues, curcumin at a dose range of 10–20 mg/kg enhanced hippocampal neurogenesis in stressed animals. The results were comparable to imipramine, a tricyclic antidepressant. The exact mechanism of curcumin's neuroregenerative ability is not clear, but is hypothesized to prevent stress-induced decrease in serotonin 5-$HT_{1A}$ mRNA and BDNF protein levels in the hippocampus[34]. Furthermore, curcumin reversed chronic stress-induced reduction in BDNF protein levels[35]. *In vitro* studies conducted in cultured rat cortical neurons have depicted that curcumin also reversed glutamate-induced decrease in BDNF levels[36].
- **Curcumin is an anti-inflammatory agent:** Inflammation is known to play a major role in the pathophysiology of major depressive syndrome. The role of T-cell dysfunction in the pathophysiology of depression has been reported earlier[37]. There is a predominance of cytokine-producing helper T cells, type 1 (Th1) or type 2 (Th2), in major depression[37]. Curcumin is a potent anti-inflammatory compound. Curcumin inhibits the cyclooxygenase-2 (COX-2) isoenzyme. Moreover, it inhibits transcription of nuclear factor-$\kappa$B (NF-$\kappa$B)[38]. Curcumin blocked the synthesis of inducible nitric oxide synthase (NOS) enzyme, thus reducing the release of inflammatory NO[39]. In one study, curcumin was found to lower the levels of interleukin (IL)-1$\beta$ cytokine by approximately 60%[40]. Further, curcumin at lower doses reduced the expression of inflammatory markers of astroglial cells[40]. The anti-inflammatory activity of curcumin is comparable to the blockers of tumor necrosis factor-alpha (TNF-$\alpha$)[1]. Therefore, based on the above evidences, it can be hypothesized that the anti-inflammatory property of curcumin contributes to its antidepressant activity.

The present review attempts to discuss the antidepressant property of curcumin in animal models of depression. Further, efforts have been made to understand the mechanism of curcumin's antidepressant profile in these animal models. It is strongly suggested that clinical trials must be initiated to explore its antidepressant activity.

## CURCUMIN IN ANIMAL MODELS OF DEPRESSION

Curcumin has shown promising activity in animal models of depression. The antidepressant profile of curcumin has been explored in various animal models of behavioral despair described below.

### Forced Swim and Tail Suspension Tests

The forced swim and tail suspension tests are the most commonly employed behavioral paradigms of despair. Although these behavioral models do not mimic the human state of major depression, they are the test models used to screen antidepressant molecules. Curcumin at a dose range of 5–10 mg/kg (p.o.) decreased the immobility period in both forced swim and tail suspension tests depicting antidepressant-like activity. These doses did not have any effect on the locomotor activity in mice[41]. In one of the studies carried out in our laboratory, curcumin (10–80 mg/kg., i.p.) decreased the immobility period in a mouse forced swim test, the maximum anti-immobility effect was reached after 90 min of its administration[32]. Curcumin at doses of 40 and 80 mg/kg also reversed the reserpine-induced behavioral despair in mice. The exact mechanism of antidepressant action of curcumin is not known, but is speculated to act through inhibiting the activity of both MAO-A and MAO-B enzymes. The MAO-inhibiting property of curcumin is further confirmed from a study in which it enhanced the anti-immobility effect of tranylcypromine (nonselective MAO inhibitor; 5 mg/kg, i.p.), and selegiline (MAO-B inhibitor; 5 mg/kg, i.p.) in mouse forced swim test[32].

Curcumin (20 mg/kg) further potentiated the antidepressant-like activity of subeffective doses of venlafaxine (dual reuptake inhibitor of serotonin and norepinephrine; 4 mg/kg, i.p.), fluoxetine (selective serotonin reuptake inhibitor; 5 mg/kg, i.p.), and bupropion (dopamine reuptake inhibitor; 5 mg/kg., i.p.)[32] (Fig. 3). However, curcumin did not potentiate the antidepressant activity of desipramine (5 mg/kg, i.p.)[32] (Fig. 3). This suggests that the antidepressant action of curcumin involves the participation of serotoninergic and dopaminergic components, but not the noradrenergic system.

A study involving the measurement of alterations in neurotransmitter levels in mice revealed an increase in the levels of serotonin and dopamine following curcumin administration[32] (Fig. 4). The levels of norepinephrine were not altered. Further, when administered simultaneously with serotoninergic agents, curcumin resulted in a more pronounced increase in the levels of serotonin as compared to the effect *per se*. Wang and colleagues demonstrated that the antidepressant action of curcumin is blocked by p-chlorophenylalanine (a tryptophan hydroxylase inhibitor that depletes the serotonin levels)[42], suggesting the major involvement of serotonin neurotransmitter in the antidepressant activity of curcumin. Moreover, it was concluded that the antidepressant action of curcumin involves the participation of 5-$HT_{1A/1B}$ and 5-$HT_{2C}$ receptors[42]. However, in another study conducted by Xu et al., an increase in the levels of noradrenaline was found in the frontal cortex and striatum regions of the mouse brain following curcumin administration[33].

The use of curcumin as a drug has always been questioned due to its poor oral bioavailability. However, there are various methods proposed to improve the bioavailability of curcumin. One of the methods has been tested in our laboratory, in which curcumin was administered along with piperine (bioavailability-enhancing agent). It has been demonstrated that piperine enhances the antidepressant-like activity of curcumin in mouse forced swim test[32] and unpredictable mild chronic stress[43].

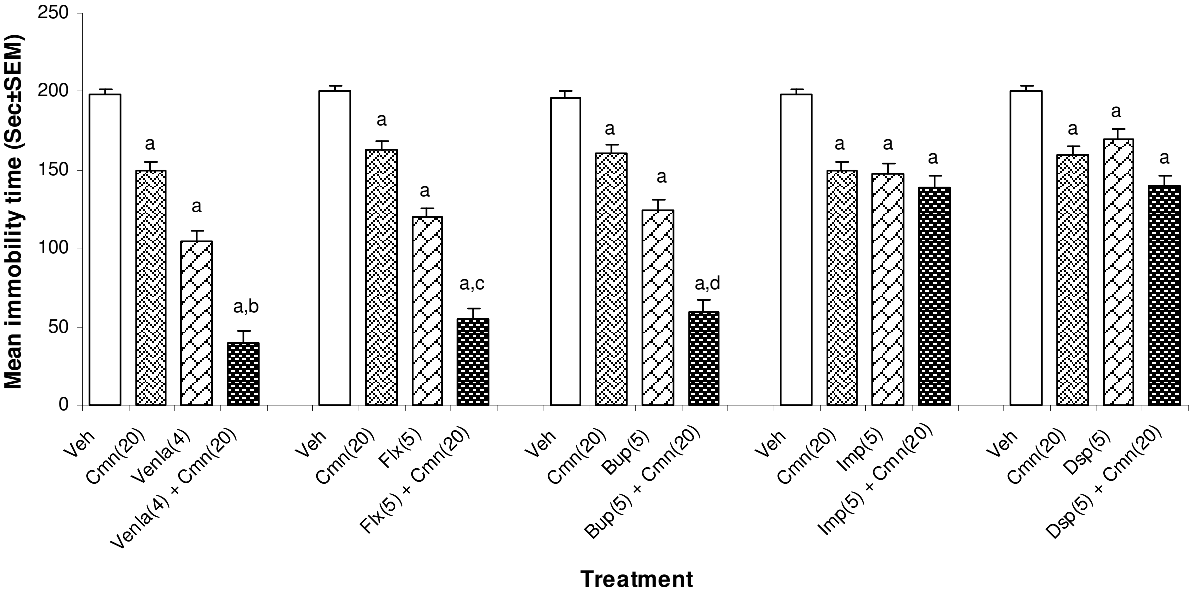

**FIGURE 3.** Effect of the combination of a subthreshold dose of curcumin (20 mg/kg., i.p.) with subthreshold doses of known antidepressants (venlafaxine, fluoxetine, imipramine, desipramine, and bupropion) on immobility period. a = $p \leq 0.05$ as compared with vehicle group, b = $p \leq 0.05$ as compared with curcumin (20) and venlafaxine (4), c = $p \leq 0.05$ as compared with curcumin (20) and fluoxetine (5), d = $p \leq 0.05$ as compared with curcumin (20) and bupropion (5). (Reproduced from Kulkarni et al.[32]. With permission.)

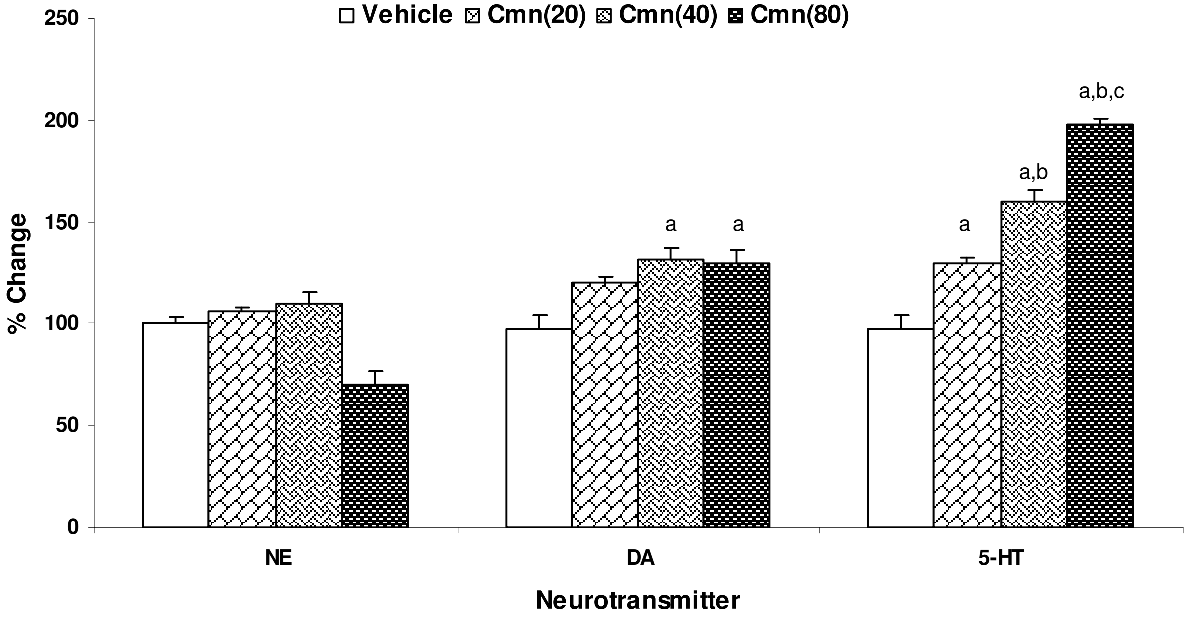

**FIGURE 4.** Effect of acute administration of curcumin (20–80 mg/kg., i.p.) on brain neurotransmitter levels. a = $p \leq 0.05$ as compared to vehicle, b = $p \leq 0.05$ as compared to curcumin (20 mg/kg), c = $p \leq 0.05$ as compared to curcumin (40 mg/kg) treated groups. (Reproduced from Kulkarni et al.[32]. With permission.)

## Unpredictable Mild Stress Model of Depression

Chronic administration of various uncontrollable stressful conditions, a procedure known as “chronic unpredictable stress”, is an appropriate model for the preclinical evaluation of antidepressants[44]. In one of the studies carried out in our laboratory, we explored the effect of curcumin in this unpredictable stress model of depression in rats[43]. In brief, rats were subjected to an unpredictable stress paradigm once a day over a period of 21 days[43]. Chronically stressed rats exhibited a significant increase in the immobility period as compared to control animals in a forced swim test. This immobility period was reversed by daily administration of curcumin (20 and 40 mg/kg, i.p.)[43]. Further, piperine potentiated the antidepressant-like effect of curcumin in this test model. Also, chronic stress for 21 days increased the activity of MAO enzymes, resulting in the depletion of the neurotransmitter levels. Interestingly, this enhanced MAO activity was brought back to normal by curcumin treatment[43]. It was further tested that chronic unpredictable stress depleted the levels of norepinephrine, serotonin, and dopamine in the whole brain. Chronic administration of curcumin normalized the depleted levels of serotonin and dopamine without affecting norepinephrine[43]. This is similar to our early hypothesis that curcumin does not have any effect on the noradrenergic neurotransmission.

Li and colleagues showed that curcumin administration reversed the unpredictable stress-induced decrease in consumption of sucrose in rats[45]. Further, curcumin was effective in reversing the stress-induced decrease in serum corticosterone levels. The antistress effect of curcumin was hypothesized to involve the adenyl cyclase/cyclic adenosine monophosphate pathways[45]. This is also evident from the fact that curcumin enhanced the AMP response element binding protein in the hippocampus, cortex, and hypothalamus regions of the rats who were challenged with chronic unpredictable mild stress[45].

Xu and colleagues recently demonstrated that rats subjected to chronic stress for 20 days displayed poor performance in the shuttle-box task[33]. Moreover, an abnormal weight ratio of adrenal gland to whole body, increased corticosterone levels in the serum, and reduced glucocorticoid receptor mRNA expression were detected in chronically stressed animals[33]. All these alterations induced by chronic unpredictable stress were reversed by prior treatment of curcumin.

## Olfactory Bulbectomy

Another reliable technique for screening antidepressant molecules has been the bilateral removal of the olfactory bulbs. This procedure elicits a variety of behavioral, neurochemical, neuroendocrine, and neuroimmune alterations, many of which mimic the symptoms of depression[46]. Curcumin has been found to be effective in the olfactory bulbectomy model of depression. Curcumin reversed olfactory bulbectomy–induced behavioral abnormalities, such as hyperactivity, in the open field and deficits in step-down passive avoidance. Curcumin also reversed the olfactory bulbectomy–induced decrease in norepinephrine, serotonin, and dopamine levels in the frontal cortex regions of the rat brain[41]. Similarly, the olfactory bulbectomy–induced decrease in the levels of serotonin, noradrenaline, 5-hydroxyindoleacetic acid, and dihydroxyphenylacetic acid in the rat hippocampus region was reversed by chronic administration of curcumin[41].

## Chronic Fatigue Model

Curcumin has been found to attenuate various behavioral and biochemical alterations induced by chronic fatigue. Chronic fatigue represents one of the core symptoms of depression. In one of the studies, animals exposed to chronic fatigue demonstrated an increase in the immobility period in the forced swim test, which was reversed by daily administration of curcumin (5–60 mg/kg). Further, curcumin attenuated chronic fatigue–induced alterations in various oxidative stress parameters, such as enhanced lipid peroxidation, nitrite, and TNF-$\alpha$, and reduced glutathione levels[47], thus showing the antioxidant property.

## FUTURE PROSPECTS

Notwithstanding the availability of several new synthetic molecules generated through advanced new drug development procedures, the natural products have had their own identity and efficacy in alleviating human suffering since ancient times. Curcumin is one such fabulous molecule that has been described in the ancient medical writings (Ayurveda) of India. It is one of the most prevalent household remedies still in use in both rural and urban India. The modern scientific approaches have demonstrated the therapeutic potential of curcumin in various diseases, such as cancer, cognitive impairment, colitis, etc. Despite the availability of vast preclinical evidence in support of the antidepressant effect of curcumin, human testing is necessary to bring its antidepressant activity into focus. Several programmed screening approaches may be required to rule out the exact mechanism of action of curcumin in alleviating the symptoms of depression. Also, testing for synergistic strategies of curcumin with the existing antidepressants in clinical setup may result in a new pace in the process of drug development for depression in humans. The dietary repercussions of curcumin intake in relation to the prevalence of depression and related mood disorders should also be strictly monitored in persons especially prone to familial depression.

## CONCLUSION

The preclinical evidence put forward in this review accounts for the antidepressant activity of curcumin and thus adds a new dimension in its vast therapeutic benefits. The molecule is known to modulate the neurotransmitter levels in the brain and increases the neurotrophic factors, thus further enhancing neuronal survival. The positive results foreseen in the experimental studies necessitate the testing of this natural remedy in humans in order to advance the antidepressant drug development process.

## References

1. Aggarwal, B.B., Sundaram, C., Malani, N., and Ichikawa, H. (2007) Curcumin: the Indian solid gold. *Adv. Exp. Med. Biol.* **595,** 1–75.
2. Sreejayan, R. and Rao, M.N. (1994) Curcuminoids as potent inhibitors of lipid peroxidation. *J. Pharm. Pharmacol.* **46,** 1013–1016.
3. Ammon, H.P. and Wahl, M.A. (1991) Pharmacology of Curcuma longa. *Planta Med*. **57,** 1–7.
4. Brouet, I. and Ohshima, H. (1995) Curcumin, an anti-tumour promoter and anti-inflammatory agent, inhibits induction of nitric oxide synthase in activated macrophages. *Biochem. Biophys. Res. Commun*. **206,** 533–540.
5. Dikshit, M., Rastogi, L., Shukla, R., and Srimal, R.C. (1995) Prevention of ischaemia-induced biochemical changes by curcumin and quinidine in the cat heart. *Indian J. Med. Res.* **101,** 31–35.
6. Johnson, S.M., Gulhati, P., Arrieta, I., Wang, X., Uchida, T., Gao, T., and Evers, B.M. (2009) Curcumin inhibits proliferation of colorectal carcinoma by modulating Akt/mTOR signaling. *Anticancer Res*. **29,** 3185–3190.
7. Limtrakul, P., Lipigorngoson, S., Namwong, O., Apisariyakul, A., and Dunn, F.W. (1997) Inhibitory effect of dietary curcumin on skin carcinogenesis in mice. *Cancer Lett.* **116,** 197–203.
8. Kiso, Y., Suzuki, Y., Watanabe, N., Oshima, Y., and Hikino, H. (1983) Antihepatotoxic principles of Curcuma longa rhizomes. *Planta Med*. **49,** 185–187.
9. Rao, C.V., Rivenson, A., Simi, B., and Reddy, B.S. (1995) Chemoprevention of colon carcinogenesis by dietary curcumin, a naturally occurring plant phenolic compound. *Cancer Res*. **55,** 259–266.
10. Srinivasan, M. (1972) Effect of curcumin on blood sugar as seen in a diabetic subject. *Indian J. Med. Sci*. **26,** 269–270.
11. Babu, P.S. and Srinivasan, K. (1995) Influence of dietary curcumin and cholesterol on the progression of experimentally induced diabetes in albino rat. *Mol. Cell. Biochem*. **152,** 13–21.
12. Arun, N. and Nalini, N. (2002) Efficacy of turmeric on blood sugar and polyol pathway in diabetic albino rats. *Plant Foods Hum. Nutr*. **57,** 41–52.
13. Srivastava, R., Dikshit, M., Srimal, R.C., and Dhawan, B.N. (1985) Antithrombotic effect of curcumin. *Thromb. Res.* **40,** 413–417.
14. Deodhar, S.D., Sethi, R., and Srimal, R.C. (1980) Preliminary study on antirheumatic activity of curcumin (diferuloyl methane). *Indian J. Med. Res*. **71,** 632–634.

15. Ma, Q.L., Yang, F., Rosario, E.R., Ubeda, O.J., Beech, W., Gant, D.J., Chen, P.P., Hudspeth, B., Chen, C., Zhao, Y., Vinters, H.V., Frautschy, S.A., and Cole, G.M. (2009) Beta-amyloid oligomers induce phosphorylation of tau and inactivation of insulin receptor substrate via c-Jun N-terminal kinase signaling: suppression by omega-3 fatty acids and curcumin. *J. Neurosci*. **29,** 9078–9089.
16. Jagatha, B., Mythri, R.B., Vali, S., and Bharath, M.M. (2008) Curcumin treatment alleviates the effects of glutathione depletion in vitro and in vivo: therapeutic implications for Parkinson's disease explained via in silico studies. *Free Radic. Biol. Med.* **44,** 907–917.
17. Bishnoi, M., Chopra, K., and Kulkarni, S.K. (2008) Protective effect of Curcumin, the active principle of turmeric (Curcuma longa) in haloperidol-induced orofacial dyskinesia and associated behavioural, biochemical and neurochemical changes in rat brain. *Pharmacol. Biochem. Behav*. **88,** 511–522.
18. Jagota, A. and Reddy, M.Y. (2007) The effect of curcumin on ethanol induced changes in suprachiasmatic nucleus (SCN) and pineal. *Cell. Mol. Neurobiol*. **27,** 997–1006.
19. Riemer, C., Burwinkel, M., Schwarz, A., Gültner, S., Mok, S.W., Heise, I., Holtkamp, N., and Baier, M. (2008) Evaluation of drugs for treatment of prion infections of the central nervous system. *J. Gen. Virol*. **89,** 594–597.
20. Ovbiagele, B. (2008) Potential role of curcumin in stroke prevention. *Expert Rev Neurother.* **8,** 1175–1176.
21. Kumar, A., Dogra, S., and Prakash, A. (2009) Protective effect of curcumin (Curcuma longa), against aluminium toxicity: possible behavioral and biochemical alterations in rats. *Behav. Brain Res*. **205(2),** 384–390.
22. Jyoti, A., Sethi, P., and Sharma, D. (2009) Curcumin protects against electrobehavioral progression of seizures in the iron-induced experimental model of epileptogenesis. *Epilepsy Behav.* **14,** 300–308.
23. Sharma, S., Chopra, K., and Kulkarni, S.K. (2007) Effect of insulin and its combination with resveratrol or curcumin in attenuation of diabetic neuropathic pain: participation of nitric oxide and TNF-alpha. *Phytother. Res*. **21,** 278–283.
24. Wong, M.L. and Licinio, J. (2001) Research and treatment approaches to depression. *Nat. Rev. Neurosci.* **2,** 343–351.
25. Wong, M.L. and Licinio, J. (2004) From monoamines to genomic targets: a paradigm shift for drug discovery in depression. *Nat. Rev. Drug Discov.* **3,** 136–151.
26. Kessler, R.C., Berglund, P., Demler, O., Jin, R., Koretz, D., Merikangas, K.R., Rush, A.J., Walters, E.E., and Wang, P.S. (2003) National Comorbidity Survey Replication. The epidemiology of major depressive disorder: results from the National Comorbidity Survey Replication (NCS-R). *JAMA* **289,** 3095–4105.
27. Greist, J.H., Mundt, J.C., and Kobak, K. (2002) Factors contributing to failed trials of new agents: can technology prevent some problems? *J. Clin. Psychiatry* **2,** 8–13.
28. Artigas, F., Romero, L., de Montigny, C., and Blier, P. (1996) Acceleration of the effect of selected antidepressant drugs in major depression by 5-HT1A antagonists. *Trends Neurosci.* **19,** 378–383.
29. Ballard, C., Bannister, C., Solis, M., Oyebode, F., and Wilcock, G. (1996) The prevalence, associations and symptoms of depression amongst dementia sufferers. *J. Affect. Disord.* **36,** 135–144.
30. Stepaniuk, J., Ritchi, L., and Tuokko, H.A. (2008) Neuropsychiatric impairments as predictors of mild cognitive impairment, dementia, and Alzheimer's disease. *Am. J. Alzheimers Dis .Other Demen.* **23,** 326–333.
31. Kokmen, E., Beard, C.M., O'Brien, P.C., and Kurland, L.T. (1996) Epidemiology of dementia in Rochester, Minnesota. *Mayo Clin. Proc*. **71**, 275–282.
32. Kulkarni, S.K., Bhutani, M.K., and Bishnoi, M. (2008) Antidepressant activity of curcumin: involvement of serotonin and dopamine system. *Psychopharmacology (Berl.)* **201,** 435–442.
33. Xu, Y., Ku, B.S., Yao, H.Y., Lin, Y.H., Ma, X., Zhang, Y.H., and Li, X.J. (2005) The effects of curcumin on depressive-like behaviors in mice. *Eur. J Pharmacol.* **518,** 40–46.
34. Xu, Y., Ku, B., Cui, L., Li, X., Barish, P.A., Foster, T.C., and Ogle, W.O. (2007). Curcumin reverses impaired hippocampal neurogenesis and increases serotonin receptor 1A mRNA and brain-derived neurotrophic factor expression in chronically stressed rats. *Brain Res.* **1162,** 9–18.
35. Xu, Y., Ku, B., Tie, L., Yao, H., Jiang, W., Ma, X., and Li, X. (2006) Curcumin reverses the effects of chronic stress on behavior, the HPA axis, BDNF expression and phosphorylation of CREB. *Brain Res*. **1122,** 56–64.
36. Wang, R., Li, Y.B., Li, Y.H., Xu, Y., Wu, H.L., and Li, X.J. (2008) Curcumin protects against glutamate excitotoxicity in rat cerebral cortical neurons by increasing brain-derived neurotrophic factor level and activating TrkB. *Brain Res.* **1210,** 84–91.
37. Wong, M.L., Dong, C., Maestre-Mesa, J., and Licinio, J. (2008) Polymorphisms in inflammation-related genes are associated with susceptibility to major depression and antidepressant response. *Mol. Psychiatry* **13,** 800–812.
38. Xu, Y.X, Pindolia, K.R., Janakiraman, N., Chapman, R.A., and Gautam, S.C. (1998) Curcumin inhibits IL-1 alpha and TNF-alpha induction of AP-1 and NK-κB DNA-binding activity in bone marrow stromal cells. *Hematopathol. Mol. Hematol.* **11,** 49–62.
39. Chan, M.M., Huang, H.I., Fenton, M.R., and Fong, D. (1998) In vivo inhibition of nitric oxide synthase gene expression by curcumin, a cancer preventive natural product with anti-inflammatory properties. *Biochem. Pharmacol.* **55,** 1955–1962.
40. Lim, G.P., Chu, T., Yang, F., Beech, W., Frautschy, S.A., and Cole, G.M. (2001) The curry spice curcumin reduces oxidative damage and amyloid pathology in an Alzheimer transgenic mouse. *J. Neurosci.* **21,** 8370–8377.
41. Xu, Y., Ku, B.S., Yao, H.Y., Lin, Y.H., Ma, X., Zhang, Y.H., and Li, X.J. (2005) Antidepressant effects of curcumin in the forced swim test and olfactory bulbectomy models of depression in rats. *Pharmacol. Biochem. Behav*. **82,** 200–206.

42. Wang, R., Xu, Y., Wu, H.L., Li, Y.B., Li, Y.H., Guo, J.B., and Li, X.J. (2008)The antidepressant effects of curcumin in the forced swimming test involve 5-HT1 and 5-HT2 receptors. *Eur. J. Pharmacol.* **578,** 43–50.
43. Bhutani, M.K., Bishnoi, M., and Kulkarni, S.K. (2009) Anti-depressant like effect of curcumin and its combination with piperine in unpredictable chronic stress-induced behavioral, biochemical and neurochemical changes. *Pharmacol. Biochem. Behav*. **92,** 39–43.
44. Katz, R.J. and Schmaltz, K. (1980) Dopaminergic involvement in attention, a novel animal model. *Prog. Neuropsychopharmacol*. **4,** 585–590.
45. Li, Y.C., Wang, F.M., Pan, Y., Qiang, L.Q., Cheng, G., Zhang, W.Y., and Kong, L.D. (2009) Antidepressant-like effects of curcumin on serotonergic receptor-coupled AC-cAMP pathway in chronic unpredictable mild stress of rats. *Prog. Neuropsychopharmacol. Biol. Psychiatry* **33(3),** 435–449.
46. Webster, H.H., Flores, G., Marcotte, E.R., Cecyre, D., Quirion, R., and Srivastava, L.K. (2000) Olfactory bulbectomy alters NMDA receptor levels in the rat prefrontal cortex. *Synapse* **37,** 159–162.
47. Gupta, A., Vij, G., Sharma, S., Tirkey, N., Rishi, P., and Chopra, K. (2009) Curcumin, a polyphenolic antioxidant, attenuates chronic fatigue syndrome in murine water immersion stress model. *Immunobiology* **214,** 33–39.
